# Cyanomethanaminium tetra­fluoro­borate

**DOI:** 10.1107/S1600536810025857

**Published:** 2010-07-07

**Authors:** Meng Ting Han, Yuan Zhang

**Affiliations:** a Ordered Matter Science Research Center, College of Chemistry and Chemical Engineering, Southeast University, Nanjing 211189, People’s Republic of China

## Abstract

In the title compound, C_2_H_5_N_2_^+^·BF_4_^−^, the cations and anions are connected *via* inter­molecular N—H⋯F and C—H⋯F hydrogen bonds, forming a three-dimensional network.

## Related literature

For background to the development of ferroelectric pure organic or inorganic compounds, see: Haertling (1999 ▶); Homes *et al.* (2001 ▶). For thesynthesis of a variety of compounds with potential piezoelectric and ferroelectric properties, see: Fu *et al.* (2009 ▶); Hang *et al.* (2009 ▶). For comparison bond lengths and bond angles, see: Wishkerman & Bernstein (2006 ▶).
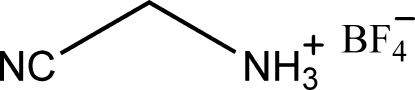


## Experimental

### 

#### Crystal data

C_2_H_5_N_2_^+^·BF_4_^−^
*M* *_r_* = 143.89
Orthorhombic, 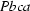

*a* = 9.790 (2) Å
*b* = 10.204 (2) Å
*c* = 11.057 (2) Å
*V* = 1104.6 (4) Å^3^
*Z* = 8
Mo *K*α radiation
μ = 0.20 mm^−1^
*T* = 293 K
0.20 × 0.20 × 0.20 mm

#### Data collection

Rigaku Mercury2 diffractometer
Absorption correction: multi-scan (*CrystalClear*; Rigaku, 2005 ▶) *T*_min_ = 0.815, *T*_max_ = 1.000
8605 measured reflections
969 independent reflections
891 reflections with *I* > 2σ(*I*)
*R*_int_ = 0.046

#### Refinement

*R*[*F* ^2^ > 2σ(*F* ^2^)] = 0.034
*wR*(*F* ^2^) = 0.097
*S* = 0.74
969 reflections
95 parameters
H atoms treated by a mixture of independent and constrained refinement
Δρ_max_ = 0.21 e Å^−3^
Δρ_min_ = −0.19 e Å^−3^

### 

Data collection: *CrystalClear* (Rigaku, 2005 ▶); cell refinement: *CrystalClear*; data reduction: *CrystalClear*; program(s) used to solve structure: *SHELXS97* (Sheldrick, 2008 ▶); program(s) used to refine structure: *SHELXL97* (Sheldrick, 2008 ▶); molecular graphics: *SHELXTL* (Sheldrick, 2008 ▶); software used to prepare material for publication: *SHELXTL*.

## Supplementary Material

Crystal structure: contains datablocks I, global. DOI: 10.1107/S1600536810025857/pv2302sup1.cif

Structure factors: contains datablocks I. DOI: 10.1107/S1600536810025857/pv2302Isup2.hkl

Additional supplementary materials:  crystallographic information; 3D view; checkCIF report

## Figures and Tables

**Table 1:** Hydrogen-bond geometry (Å, °)

*D*—H⋯*A*	*D*—H	H⋯*A*	*D*⋯*A*	*D*—H⋯*A*
C1—H1*A*⋯F2^i^	0.97	2.53	3.474 (2)	166
C1—H1*B*⋯F1^ii^	0.97	2.45	3.407 (2)	169
N2—H2*A*⋯F3	0.89 (2)	1.97 (2)	2.850 (2)	169 (2)
N2—H2*B*⋯F1^iii^	0.91 (3)	2.03 (3)	2.863 (2)	152 (2)
N2—H2*C*⋯F4^iv^	0.87 (3)	2.04 (3)	2.844 (2)	154 (2)

Symmetry codes: (i) 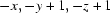; (ii) 
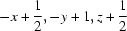
; (iii) 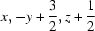; (iv) 
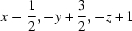
.

**Fig. 1.**
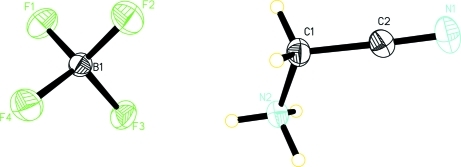
Perspective structure of the title compound, showing the atom-numbering scheme. Displacement ellipsoids are drawn at the 30% probability level.

**Fig. 2.**
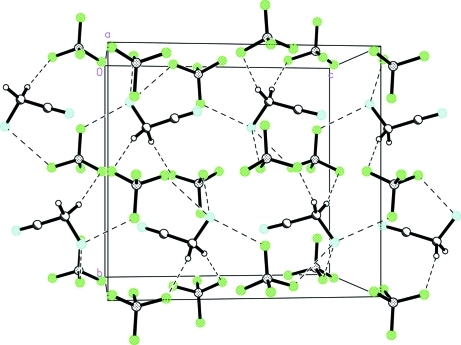
The crystal packing of the title compound viewed along the a axis showing the hydrogen bondings network.

## supplementary crystallographic information

### Comment

At present, much attention in ferroelectric material field is focused on developing ferroelectric pure organic or inorganic compounds (Haertling, 1999; Homes *et al.*, 2001). Recently we have reported the synthesis of a variety of compounds (Fu *et al.*, 2009; Hang *et al.*, 2009), which have potential piezoelectric and ferroelectric properties. In order to find more dielectric ferroelectric materials, we have investigate the physical properties of the title compound. The dielectric constant of the title compound as a function of temperature indicates that the permittivity is basically temperature-independent (dielectric constant equaling to 3.6 to 4.7), suggesting that this compound should not be ferroelectric or there may be no distinct phase transition within the measured temperature range. Similarly, below the melting point (453 K) of the compound, the dielectric constant as a function of temperature also goes smoothly, and there is no dielectric anomaly observed (dielectric constant equaling to 3.6 to 4.7). Herein, we report the synthesis and crystal structure of the title compound.

The molecular structure of the title compund is presented in Fig. 1. The bond lengths and angles are within their normal ranges (Wishkerman & Bernstein, 2006). The cations and anions are connected *via* intermolecular N—H···F and C—H···F hydrogen bonds, forming a three dimensional network (Tab. 1 & Fig. 2).

### Experimental

A mixture of aminoacetonitrile hydrochloride (0.095 g, 0.01 mol) and tetrafluoro-borate sodium (1.10 g, 0.01 mol) in water (20 ml) was stirred until clear. After several days, colourless prismatic crystals of the title compound were formed which were suitable for X-ray analysis.

### Refinement

The methylene H-atoms were positioned geometrically and refined using a riding model, with C—H = 0.97 Å and *U*_iso_(H) = 1.2_eq_(C). The H-atoms bonded to the N-atom were located from a difference map and were allowed to refine freely.

### Crystal data

**Table:**

C_2_H_5_N_2_^+^·BF_4_^−^	*F*(000) = 576
*M**_r_* = 143.89	*D*_x_ = 1.730 Mg m^−^^3^
Orthorhombic, *P**b**c**a*	Mo *K*α radiation, λ = 0.71073 Å
Hall symbol: -P 2ac 2ab	Cell parameters from 970 reflections
*a* = 9.790 (2) Å	θ = 2.6–25.0°
*b* = 10.204 (2) Å	µ = 0.20 mm^−^^1^
*c* = 11.057 (2) Å	*T* = 293 K
*V* = 1104.6 (4) Å^3^	Prism, colorless
*Z* = 8	0.20 × 0.20 × 0.20 mm

### Data collection

**Table:**

Rigaku Mercury2 (2x2 bin mode) diffractometer	969 independent reflections
Radiation source: fine-focus sealed tube	891 reflections with *I* > 2σ(*I*)
graphite	*R*_int_ = 0.046
Detector resolution: 13.6612 pixels mm^-1^	θ_max_ = 25.0°, θ_min_ = 3.4°
CCD_Profile_fitting scans	*h* = −11→11
Absorption correction: multi-scan (*CrystalClear*; Rigaku, 2005)	*k* = −12→12
*T*_min_ = 0.815, *T*_max_ = 1.000	*l* = −13→13
8605 measured reflections	

### Refinement

**Table:**

Refinement on *F*^2^	Secondary atom site location: difference Fourier map
Least-squares matrix: full	Hydrogen site location: inferred from neighbouring sites
*R*[*F*^2^ > 2σ(*F*^2^)] = 0.034	H atoms treated by a mixture of independent and constrained refinement
*wR*(*F*^2^) = 0.097	*w* = 1/[σ^2^(*F*_o_^2^) + (0.0711*P*)^2^ + 0.9831*P*] where *P* = (*F*_o_^2^ + 2*F*_c_^2^)/3
*S* = 0.74	(Δ/σ)_max_ < 0.001
969 reflections	Δρ_max_ = 0.21 e Å^−^^3^
95 parameters	Δρ_min_ = −0.19 e Å^−^^3^
0 restraints	Extinction correction: *SHELXL97* (Sheldrick, 2008), Fc^*^=kFc[1+0.001xFc^2^λ^3^/sin(2θ)]^-1/4^
Primary atom site location: structure-invariant direct methods	Extinction coefficient: 0.034 (4)

### Special details

**Table:**

Geometry. All e.s.d.'s (except the e.s.d. in the dihedral angle between two l.s. planes) are estimated using the full covariance matrix. The cell e.s.d.'s are taken into account individually in the estimation of e.s.d.'s in distances, angles and torsion angles; correlations between e.s.d.'s in cell parameters are only used when they are defined by crystal symmetry. An approximate (isotropic) treatment of cell e.s.d.'s is used for estimating e.s.d.'s involving l.s. planes.
Refinement. Refinement of *F*^2^ against ALL reflections. The weighted *R*-factor *wR* and goodness of fit *S* are based on *F*^2^, conventional *R*-factors *R* are based on *F*, with *F* set to zero for negative *F*^2^. The threshold expression of *F*^2^ > σ(*F*^2^) is used only for calculating *R*-factors(gt) *etc*. and is not relevant to the choice of reflections for refinement. *R*-factors based on *F*^2^ are statistically about twice as large as those based on *F*, and *R*- factors based on ALL data will be even larger.

### Fractional atomic coordinates and isotropic or equivalent isotropic displacement parameters (Å^2^)

**Table:**

	*x*	*y*	*z*	*U*_iso_*/*U*_eq_	
F1	0.20443 (11)	0.51691 (10)	0.27959 (10)	0.0507 (4)	
F3	0.25530 (11)	0.69102 (10)	0.39731 (9)	0.0482 (4)	
F4	0.39397 (10)	0.51360 (11)	0.39567 (11)	0.0536 (4)	
N2	0.11384 (16)	0.80444 (15)	0.59675 (13)	0.0363 (4)	
F2	0.18692 (13)	0.50016 (11)	0.48270 (11)	0.0586 (4)	
C1	0.05747 (17)	0.69059 (16)	0.66197 (16)	0.0380 (4)	
H1A	−0.0034	0.6428	0.6086	0.046*	
H1B	0.1315	0.6323	0.6844	0.046*	
C2	−0.01713 (15)	0.72917 (17)	0.77058 (14)	0.0363 (4)	
N1	−0.07586 (17)	0.75568 (18)	0.85538 (14)	0.0553 (5)	
B1	0.25945 (17)	0.55466 (18)	0.38936 (15)	0.0305 (4)	
H2C	0.052 (3)	0.861 (3)	0.574 (2)	0.079 (8)*	
H2B	0.170 (3)	0.852 (2)	0.645 (2)	0.079 (8)*	
H2A	0.165 (3)	0.778 (2)	0.535 (2)	0.070 (7)*	

### Atomic displacement parameters (Å^2^)

**Table:**

	*U*^11^	*U*^22^	*U*^33^	*U*^12^	*U*^13^	*U*^23^
F1	0.0617 (7)	0.0499 (6)	0.0406 (6)	−0.0010 (5)	−0.0146 (5)	−0.0072 (4)
F3	0.0601 (7)	0.0311 (6)	0.0535 (7)	0.0006 (4)	0.0087 (5)	−0.0054 (4)
F4	0.0367 (6)	0.0529 (7)	0.0713 (8)	0.0075 (4)	−0.0063 (5)	−0.0017 (5)
N2	0.0375 (8)	0.0398 (8)	0.0314 (8)	0.0024 (6)	0.0054 (6)	0.0001 (6)
F2	0.0686 (8)	0.0589 (7)	0.0483 (7)	−0.0096 (6)	0.0185 (6)	0.0086 (5)
C1	0.0442 (9)	0.0329 (8)	0.0369 (9)	0.0026 (6)	0.0089 (7)	−0.0006 (7)
C2	0.0333 (8)	0.0423 (9)	0.0333 (9)	0.0022 (7)	0.0001 (7)	0.0032 (7)
N1	0.0533 (9)	0.0722 (12)	0.0404 (9)	0.0060 (8)	0.0127 (7)	−0.0008 (9)
B1	0.0320 (9)	0.0299 (9)	0.0296 (9)	−0.0005 (7)	0.0005 (7)	−0.0006 (6)

### Geometric parameters (Å, °)

**Table:**

F1—B1	1.3828 (19)	N2—H2A	0.89 (3)
F3—B1	1.395 (2)	F2—B1	1.371 (2)
F4—B1	1.384 (2)	C1—C2	1.460 (2)
N2—C1	1.475 (2)	C1—H1A	0.9700
N2—H2C	0.87 (3)	C1—H1B	0.9700
N2—H2B	0.91 (3)	C2—N1	1.133 (2)
			
C1—N2—H2C	113.4 (17)	N2—C1—H1B	109.2
C1—N2—H2B	111.3 (16)	H1A—C1—H1B	107.9
H2C—N2—H2B	104 (2)	N1—C2—C1	178.16 (19)
C1—N2—H2A	110.3 (16)	F2—B1—F1	110.25 (14)
H2C—N2—H2A	112 (2)	F2—B1—F4	109.41 (14)
H2B—N2—H2A	106 (2)	F1—B1—F4	109.31 (14)
C2—C1—N2	112.16 (14)	F2—B1—F3	110.01 (13)
C2—C1—H1A	109.2	F1—B1—F3	108.78 (13)
N2—C1—H1A	109.2	F4—B1—F3	109.06 (13)
C2—C1—H1B	109.2		

### Hydrogen-bond geometry (Å, °)

**Table:**

*D*—H···*A*	*D*—H	H···*A*	*D*···*A*	*D*—H···*A*
C1—H1A···F2^i^	0.97	2.53	3.474 (2)	166
C1—H1B···F1^ii^	0.97	2.45	3.407 (2)	169
N2—H2A···F3	0.89 (2)	1.97 (2)	2.850 (2)	169 (2)
N2—H2B···F1^iii^	0.91 (3)	2.03 (3)	2.863 (2)	152 (2)
N2—H2C···F4^iv^	0.87 (3)	2.04 (3)	2.844 (2)	154 (2)

Symmetry codes: (i) −*x*, −*y*+1, −*z*+1; (ii) −*x*+1/2, −*y*+1, *z*+1/2; (iii) *x*, −*y*+3/2, *z*+1/2; (iv) *x*−1/2, −*y*+3/2, −*z*+1.
